# Midlife managerial experience is linked to late life hippocampal morphology and function

**DOI:** 10.1007/s11682-016-9649-8

**Published:** 2016-11-15

**Authors:** C. Suo, N. Gates, M. Fiatarone Singh, N. Saigal, G. C. Wilson, J. Meiklejohn, P. Sachdev, H. Brodaty, W. Wen, N. Singh, B. T. Baune, M. Baker, N. Foroughi, Y. Wang, Michael J. Valenzuela

**Affiliations:** 1 School of Psychiatry, University of New South Wales, Sydney, NSW Australia; 2 Regenerative Neuroscience Group, Brain and Mind Research Institute, University of Sydney, Sydney, NSW Australia; 3 Centre for Healthy Brain Ageing (CHeBA), School of Psychiatry, University of New South Wales, Sydney, NSW Australia; 4 Brain and Mental Health Laboratory, Monash Institute of Cognitive and Clinical Neurosciences, School of Psychological Science, Monash University, Clayton, Australia; 5 Exercise Health and Performance Faculty Research Group, Faculty of Health Sciences and Sydney Medical School, The University of Sydney, Lidcombe, Australia; 6 Hebrew SeniorLife, Boston, MA USA; 7 Jean Mayer USDA Human Nutrition Research Center on Aging at Tufts University, Boston, MA USA; 8 Exercise Health and Performance Faculty Research Group, Faculty of Health Sciences, The University of Sydney, Lidcombe, Australia; 9 Neuropsychiatric Institute, Prince of Wales Hospital, Sydney, NSW Australia; 10 Dementia Collaborative Research Centre, University of New South Wales, Sydney, NSW Australia; 11 Department of Psychiatry, School of Medicine, University of Adelaide, Adelaide, South Australia Australia; 12 School of Exercise Science, Australian Catholic University, Strathfield, NSW Australia; 13 Clinical and Rehabilitation Research Group, Faculty of Health Sciences, The University of Sydney, Lidcombe, Australia; 14 Department of Medicine and the Diabetes Center, University of California, San Francisco, San Francisco, CA USA; 15 Brain and Mind Centre, 100 Mallett St Camperdown, Sydney, NSW 2050 Australia

**Keywords:** Cognitive lifestyle, Managerial experience, Hippocampal volume, Hippocampal morphometry, Hippocampal functional connectivity, Memory

## Abstract

An active cognitive lifestyle has been suggested to have a protective role in the long-term maintenance of cognition. Amongst healthy older adults, more managerial or supervisory experiences in midlife are linked to a slower hippocampal atrophy rate in late life. Yet whether similar links exist in individuals with Mild Cognitive Impairment (MCI) is not known, nor whether these differences have any functional implications. 68 volunteers from the Sydney SMART Trial, diagnosed with non-amnestic MCI, were divided into high and low managerial experience (HME/LME) during their working life. All participants underwent neuropsychological testing, structural and resting-state functional MRI. Group comparisons were performed on hippocampal volume, morphology, hippocampal seed-based functional connectivity, memory and executive function and self-ratings of memory proficiency. HME was linked to better memory function (*p* = 0.024), mediated by larger hippocampal volume (*p* = 0.025). More specifically, deformation analysis found HME had relatively more volume in the CA1 sub-region of the hippocampus (*p* < 0.05). Paradoxically, this group rated their memory proficiency worse (*p* = 0.004), a result correlated with diminished functional connectivity between the right hippocampus and right prefrontal cortex (*p* < 0.001). Finally, hierarchical regression modelling substantiated this double dissociation.

**Electronic supplementary material:**

The online version of this article (doi:10.1007/s11682-016-9649-8) contains supplementary material, which is available to authorized users.

## Background

Cognitive lifestyle refers to lifetime patterns of participation in educational, occupational, social and leisure activities and is increasingly recognised as a protective and modifiable risk factor in the development of dementia. An enriched cognitive lifestyle has been linked to a lower risk for incident dementia (M. J. Valenzuela and Sachdev 2006) (Valenzuela et al. 2011), less prospective cognitive decline (Marioni et al. 2012b), higher chances of recovery from mild impairment to cognitive normalcy (Marioni et al. 2012a), a slower rate of hippocampal atrophy (Valenzuela et al. 2008; Suo et al. 2012) and greater neuronal density in the frontal lobe (Valenzuela et al. 2012).

Previously, in a population-based study of healthy older adults we showed that the link between active cognitive lifestyle and protection from hippocampal atrophy was primarily driven by high-level managerial and supervisory experience in midlife – ex-high level managers or supervisors had significantly larger hippocampal volume and slower hippocampal atrophy rate than those who had never been in charge of others in their working life (Suo et al. 2012). This is particularly interesting because a fast rate of hippocampal atrophy or smaller hippocampal volume is a sensitive and specific predictor of dementia (Jack et al. 2000) (Risacher et al. 2010) (Frankó et al. 2013) (den Heijer et al. 2006). Yet whether the same protective association applies to individuals with dementia precursor, Mild Cognitive Impairment (MCI), is unknown.

Prediction of the relationship between occupational complexity and hippocampal structure and function in MCI from results obtained in healthy older adults is challenging. On the one hand, the neuroprotective association we previously reported (Suo et al. 2012) may continue into the preclinical phase. On the other hand, a compensatory effect can result in individuals with borderline impairment and greater exposure to complex cognitive lifestyles exhibiting more signs of disease burden (Stern et al. 1995) (Brayne et al. 2010) (Bennett et al. 2014). Indeed, previous studies have found contrasting correlations between cognitive lifestyle-related variables and brain structure/function measures in different cohorts, i.e., healthy elderly versus AD dementia (Arenaza-Urquijo et al. 2013; Sole-Padulles et al. 2009) (Seo et al. 2011) (Kidron et al. 1997) (Coffey et al. 1999). Further complicating matters, MCI has two major subtypes, amnestic MCI (aMCI) that is commonly linked to AD pathology (Albert et al. 2011), and non-amnestic MCI (naMCI) that has multiple aetiology (Petersen 2004) including both Alzheimer’s disease and cerebrovascular disease (Sudo et al. 2012). Irrespective of MCI sub-classification, diagnosis relies on both subjective and objective cognitive criteria (Petersen 2004). Cognition in one or more domains must be objectively impaired on neuropsychological tests accompanied by a subjective concern or complaint about one’s own cognitive proficiency. It is the latter that typically motivates a person to present to a medical professional and ultimately receive a diagnosis (Wong et al. 2006).

Our first objective was therefore to determine in older individuals with naMCI whether occupational supervision was related to late-life hippocampal volume and morphology based on cross-sectional data. Secondly, because links between cognition and hippocampal functional networks have been revealed by resting-state functional Magnetic Resonance Image (fMRI) (Liu et al. 2008; Wang et al. 2011), we also examined potential long-term functional implications of occupational managerial experience. Specifically, we tested whether managerial experience was linked to two of the core criteria for MCI, objective and subjective cognitive function, and further explored whether putative structural differences in the medial temporal lobe translate to subtle changes to resting-state fMRI networks. We hypothesized that like in our healthy elderly sample there would be a protective link between midlife managerial experience and hippocampal structure in late life. We also expected a difference in functional brain networks between individuals with high and low levels of managerial experience, but the direction of the difference was difficult to predict and so this was an exploratory question. Similarly, we tried to integrate hippocampal structure, function, objective memory and subjective concerns into one statistical model.

## Methods

### Participants

Participants were drawn from the Sydney SMART trial (Study of Mental Activity and Regular Training) (Gates et al. 2011b) (Suo et al. 2016), a randomised, double-blind, active-controlled longitudinal trial of resistance training and computer-based cognitive training in individuals who met ‘core criteria’ for MCI (Albert et al. 2011). All results reported here are derived from baseline data before randomization or the onset of any intervention. Participants (*N* = 100) were community-dwelling persons aged over 55, with diagnosis of MCI (self-reported memory complaint; objective cognitive deficit based on a Mini-Mental Status Examination (MMSE) score of 23-28/30 or 29/30 if error in memory registration; and no dementia (Clinical Dementia Rating of 0.5 or below). Primary exclusion criteria were clinical depression and unstable medical conditions or other neurological diseases. Full inclusion and exclusion details can be found in our published protocol (Gates et al. 2011a).

For this study further exclusion criteria were applied: 1) non-retired participants were excluded (*n* = 11) because of our specific interest on the long term correlates of managerial experience in working life on brain health after retirement (Valenzuela and Sachdev 2007); 2) *Amnestic* MCI based on an age-scaled score on the Logical Memory II WMS test of less than 5 (i.e., <=5th percentile) because aMCI was only a small proportion of our SMART cohort (*n* = 8) and exclusion therefore produced to a more homogenous naMCI sample; 3) Missed or refused MRI (*n* = 12) or severe artefacts in MRI data (*n* = 1). Our final sample with complete data comprised 68 participants. Because each neuroimaging modality is susceptible to different types of artefacts, a variable number of participants were omitted from different specific analyses as numerated below. Informed consent was obtained from all participants, in accordance with the guideline of the human research ethics committee of both the University of New South Wales and Sydney University.

### Lifetime of experience questionnaire (LEQ)

LEQ data were collected using an online system (rng.org.au/leq) in a dedicated computer room at Sydney University. During data collection, a psychologist was on hand to provide assistance and supervision. All the sub-scores and total scores were automatically calculated using the same rules as paper-based LEQ (Valenzuela and Sachdev 2007). Managerial experience is based on asking “How many people have you been in charge of?” during each five year epoch from 30 to retirement. The answer has four response options: none, 1–5 people, 6–9 people and 10 or more people. The maximum answer of any five-year period is defined as midlife managerial experience. We dichotomised the four response options into: high managerial experience (6 or more people) and low managerial experience group (less than 6 people) based on our previous observation of a non-continuous step-change in hippocampal volume in both men and women (Suo et al. 2012) and also for practical purposes based on the number of participants in these four categories. To more specifically estimate the engagement in late life, we separated out all social related questions in the late life part of LEQ. As in our previous report (Suo et al. 2012), we included non-specific questions 2–6 and social related options in non-specific question 7 and 8, and generated a late-life total social engagement subscale.

### Socio-demographic, health status, cognitive and psychological assessment

All socio-demographic and health status data were obtained by self-report using structured interviews. All cognitive and psychological data were acquired by an experienced neuropsychologist. For details of full neuropsychological battery and psychological test instruments see SMART protocol (Gates et al. 2011b). Main psychological measures are A) Self-rated Memory Appraisal, combining Subjective Memory Complaint (SMC) and Memory Awareness Rating Scale – Memory Function Scale (MARS-MFS) (Clare et al. 2002); B) Executive domain objective cognition; C) Memory domain objective cognition. Details provided in Supplementary.

### MRI

MRI data were acquired on a Philip 3 T Scanner. Details provided in Supplementary.

### Structural MRI

Left and right hippocampi were segmented using the Oxford Centre for Functional MRI of the Brian (FMRIB)’s Integrated Registration and Segmentation Tool (FIRST), according to the processing protocol of Erickson et al. (Erickson et al. 2011). Mean bilateral hippocampal volume and vertex/mesh model for each individual was calculated automatically, followed by quality checking for segmentation error, using the protocol established by the ENIGMA (Enhancing Neuro-Imaging Genetics Through Meta-Analysis) consortium. Nine (2 in HME, 7 in LME) participants were excluded from this analysis because segmentation failed due to abnormal brain structure or movement artefacts, leaving *N* = 59 for this analysis. Two group t-tests were then performed based on vertex-to-vertex analyses on the both left and right hippocampus. We used sex as a covariate, because our previous population-based study showed a significant difference of sex proportion between high and low managerial groups (Suo et al. 2012). Results were FDR corrected for multiple comparison errors. Further details provided in Supplementary.

### Resting state functional MRI

**Fig. 1:**
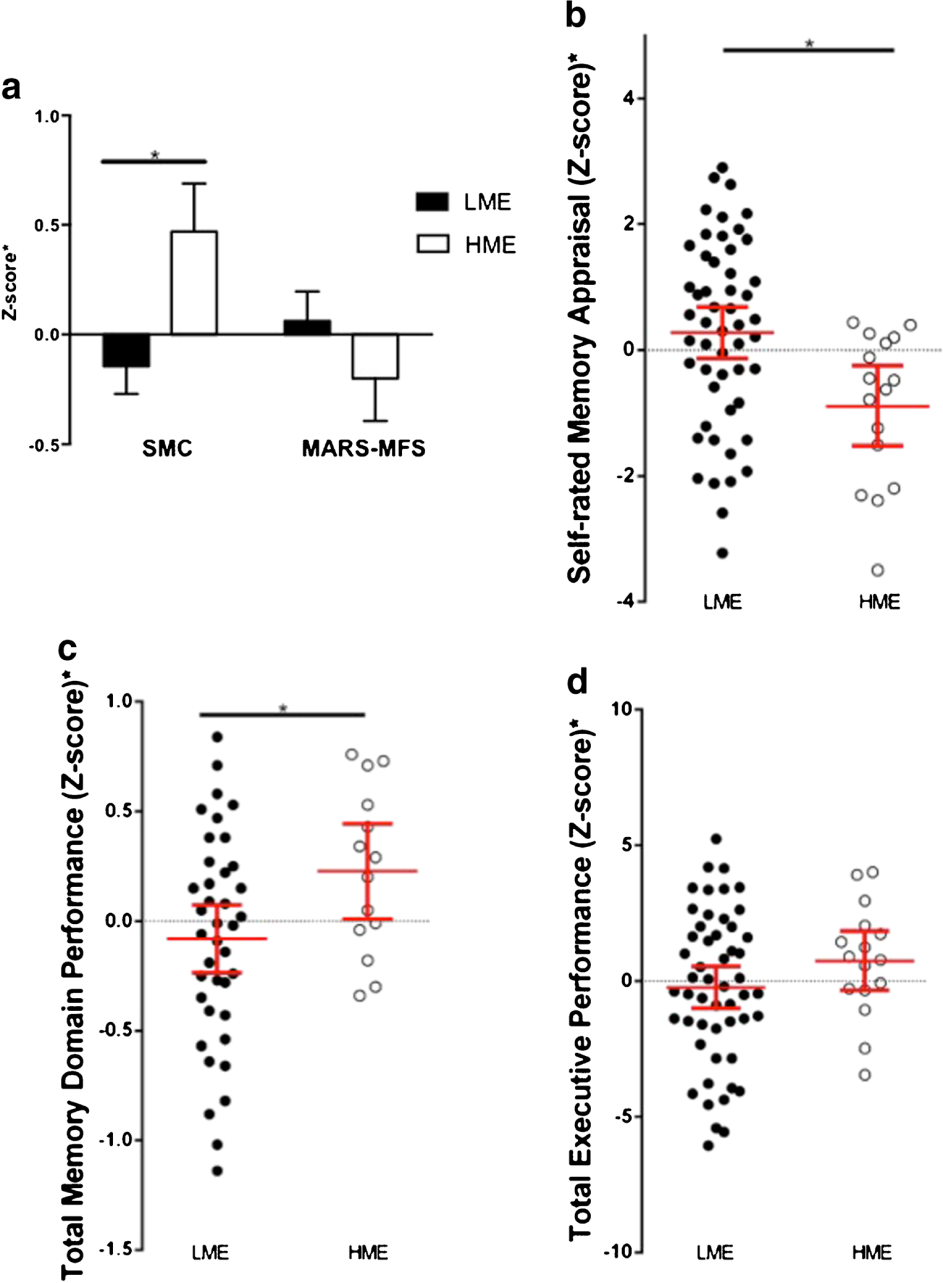
Comparison of high and low managerial experience (HME, LME) on self-rated memory appraisal, and memory domain and executive domain function. All graphs displayed adjusted means/values ± SEM. **a** HME participants had significantly more subjective memory complaints (SMC, *p* = 0.016, controlling for sex, age and education years) and tendency for lower expectations about their future memory proficiency (measured by MARS-MFS, *p* = 0.328, controlling for sex, age and education years; *p* = 0.10 unadjusted). **b** HME group showed a significantly lower overall self-rated memory domain score (*p* = 0.004, controlling for sex, age and education years). **c** HME group performed significantly better on memory tests (*p* = 0.024, after adjustment for sex, age and education years). **d** HME participants also had a trend towards better performance on tests of executive function (*p* = 0.181, after adjustment for sex, age and education years; *p* = 0.016 unadjusted)

**Fig. 2:**
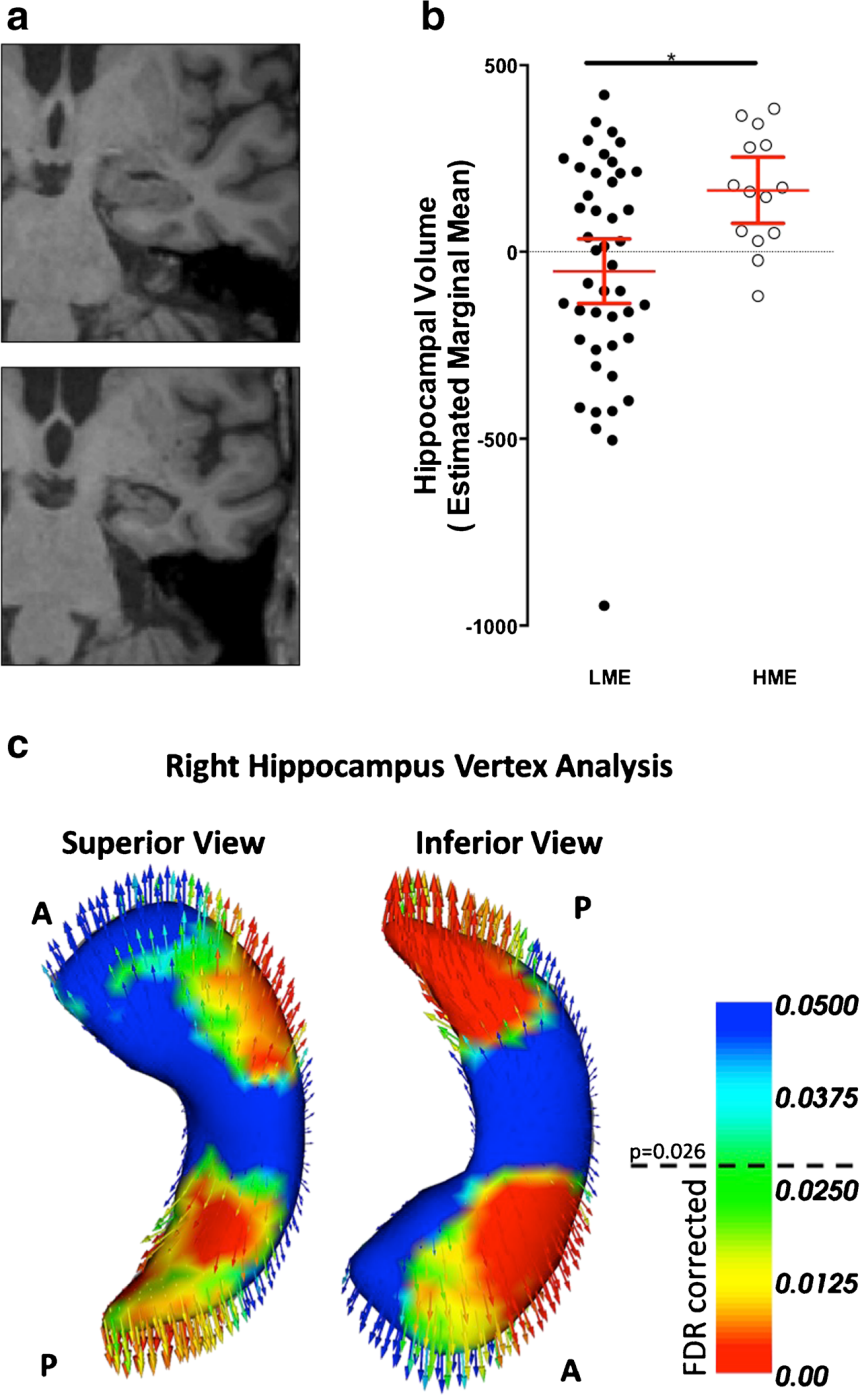
Hippocampal volume differences and vertex analysis between HME and LME groups. **a** showed visible hippocampal differences comparing a HME (*top*) and LME participant (*bottom*) at the same coronal slice. **b** HME group had larger average hippocampal volume, after adjustment for intracranial volume, sex, age and education year (*p* = 0.006). Adjusted values are estimated marginal means ± SEM after controlling for these covariances. Significance was robust to exclusion of the outliner in LME group. Vertex analysis **c** showed two main areas of morphological differences for the right hippocampus between managerial experience groups. Warm colors indicated increasing significance level where vertices with *p* < 0.026 survive FDR correction controlling for sex. *Arrows* indicated the direction of inter-group differences at each vertex, and point from the mean surface of the LME group to the HME group, indicative of relative volume inflation (i.e., larger volume in HME group at this region). A: anterior, P: posterior. See Supplementary for a 3D-rotating movie

**Fig. 3:**
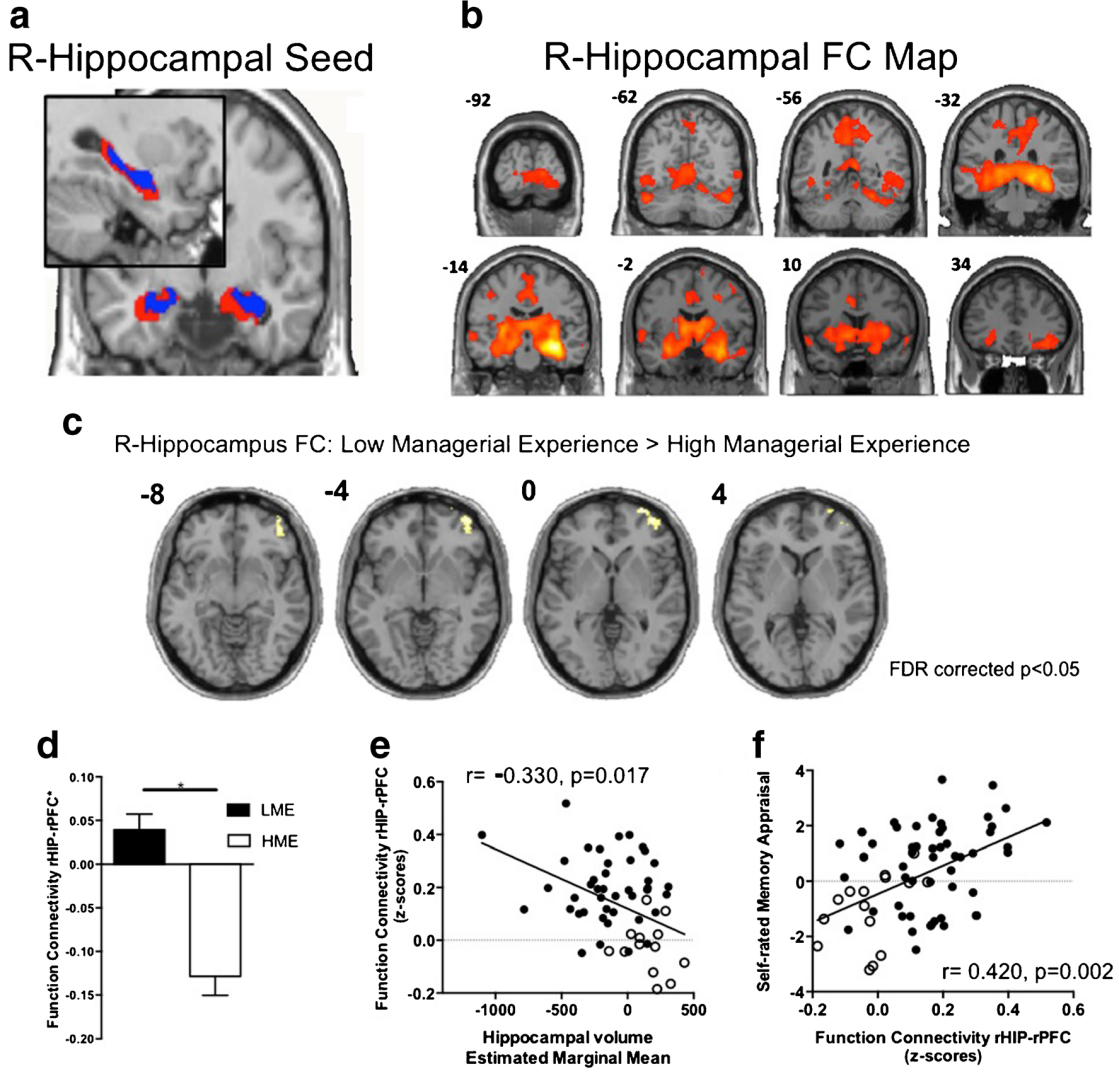
Hippocampal seed resting-state functional connectivity differences between LME and HME group. All graphs display values after adjustment for sex, age and education ± SEM. **a** Erosion of the original AAL hippocampus template (*red*) resulted in an atrophy-like template (*blue*) for use as the FC seed. **b** FC connectivity of right hippocampus, across whole sample (voxel-level p_(FWE-corr)_ = 0.000005). **c** LME group showed greater right hippocampal FC with the right middle frontal cortical gyrus (rPFC) than the HME group (k = 237; cluster-level p_(FDR-corr)_ = 0.022; controlling for sex, age and education years). **d** The rHIP-rPFC connectivity in the LME group was significantly higher than that in HME group, after controlling for sex, age and education years (F = 26.8, df = 59, *p* < 0.001). **e** rHIP-rPFC connectivity was negatively correlated with hippocampal volume, after controlling for age, sex and education year. Unfilled dots indicate HME participants. **f** Self-rated memory appraisal was positively correlated with rHIP-rPFC connectivity, after controlling for age, sex and education year. Unfilled dots indicate HME participants. NB: Right side of the image indicated the right side of brain

Four fMRI data had to be removed due to the artefacts (one participant has shunt, three discarded due to movement criteria: displacement >3 mm in any direction or rotation >5 degree along any axis) (Johnstone et al. 2006) (Kim et al. 1999), leaving *N* = 64 for fMRI analysis. We pre-processed the fMRI data and generated hippocampus seed functional connectivity (FC) map bilaterally, using SPM8 (Statistical Parametric Mapping) based tool-box (Chao-Gan and Yu-Feng 2010). Since individuals with MCI exhibited significant smaller hippocampi (Shi et al. 2009), the original Anatomical Automatic Labeling (AAL) hippocampus seed was eroded internally by 2 mm, resulting in a core hippocampal template less likely to be sensitive to partial volume effects on the BOLD signal (Figs. 1, 2 and 3a). Two group *t*-test of hippocampal functional connectivity maps was performed using SPM8, to test the topological difference between HME group and LME group, controlling for age, sex and education years. Clusters with *p* < 0.05 (cluster-level FDR correction) were consider significant, and mean functional connectivity index of these clusters were extracted for further statistical tests. Further details can be found in Supplementary.

### Statistical analysis and modelling

Zero-order structural models were first built and simplified to a final model of independent relationships using a series hierarchical linear regression models based on backwards elimination linear regressions. Where appropriate, we explicitly tested for mediation effects using the Sobel test (Preacher and Hayes 2004). More details are in Supplementary.

A p-value of <0.05 was considered indicative of statistical significance. Also, bar/scatter plots in Figs. 1, 2 and 3 displayed residual values from the regression analyses, indicating the adjusted means/values after controlling for covariates listed in legends.

## Results

### Sociodemographic profile and cognitive lifestyle

Sociodemographic and clinical characteristics are presented in Table 1. The total sample (*N* = 68) was divided into participants with low managerial experience (LME) group (*N* = 52) or high managerial experience (HME) (*N* = 16). In general, participants had mild cognitive deficits as indicated by the majority of ADAS-COG scores in the MCI range of 8–9 (Pyo et al. 2006), and negligible depressive symptoms, consistent with SMART entry criteria. Also, their overall cognitive lifestyle (LEQ = 88.4) was similar to that reported for the population-based Sydney Memory & Ageing Study (healthy aged average 93.4 (Valenzuela et al. 2013)). There were no significant differences between managerial groups based on age, MMSE, CDR, retirement year, Geriatric Depression Scale, Physical Activities Scale, total LEQ score, or any LEQ lifestage subtotal. Further, no significant group differences were found in frequency of male sex, hypertension, diabetes type II, hypercholesterolemia, income group or job profile during midlife using Chi-Square analysis. Based on our previous finding of distinct late life socialization patterns between managerial experience groups (Suo et al. 2012), we tested this again in this sample and found a significantly higher level of social engagement for the HME group compared to LME, that reduced to trend after controlling for covariates (unadjusted *p* = 0.031, F = 1.94; adjusted for age, sex and education *p* = 0.075, Table 1).

**Table 1:** Sociodemographic and clinical variables. Values represent averages ± SD unless otherwise stated

	Low Managerial Experience *N* = 52	High Managerial Experience *N* = 16	p-value*
Age	71.3 ± 6.0	68.6 ± 6.3	0.126
Gender (M/F count)*	13/39	7/9	0.210
Education (years)	12.60 ± 3.8	14.69 ± 3.2	0.052
MMSE	27.5 ± 1.4	27.9 ± 1.2	0.293
ADAS-COG	8.1 ± 3.4	6.6 ± 3.1	0.114
Clinical Dementia Rating (CDR)	0.23 ± 0.32	0.15 ± 0.40	0.429
Physical Activities Score	9.3 ± 4.0	10.7 ± 3.7	0.235
Geriatric Depression Scale	1.1 ± 1.3	1.6 ± 1.6	0.211
Hypertension (%)*	44.2	18.8	0.083
Diabetes Type II (%)*	7.7	6.3	1.000
Hypercholesterolemia (%)*	28.8	25.0	1.000
Young Adulthood Subtotal	29.3 ± 9.6	32.9 ± 7.5	0.180
Mid Life Subtotal	32.5 ± 10.3	38.6 ± 10.4	0.056
Late Life Subtotal	24.7 ± 6.2	26.3 ± 4.5	0.412
Late Life Social Engagement	7.08 ± 1.35	7.98 ± 0.86	0.031^
Total LEQ	86.2 ± 20.9	96.8 ± 16.7	0.109
Annual Income*			
Hi (>$30 K)	38.8 %	33.3 %	0.867
Mid ($15 k ~ $30 k)	26.5 %	33.3 %	
Low (<$15 K)	34.7 %	33.3 %	
Job Classification*			
Managers and Administrators	1.9 %	12.5 %	0.251
Professionals	38.5 %	62.5 %	
Tradespersons and related works	9.6 %	6.3 %	
advanced clerical and service workers	3.9 %	6.3 %	
clerical, sales and service workers	30.8 %	12.5 %	
elementary clerical, sales & service workers	9.6 %	0 %	
Labourers and related workers	5.8 %	0 %	

* Fisher’s Exact Chi-Square Test for non-parametric comparisons, otherwise T-test procedure applied

^ *p* < 0.05

### Self-rated memory proficiency

As shown in Fig. 1, participants in the HME group had significantly more memory complaints (df = 66, F = 9.3, *p* = 0.003), a trend towards lower MARS-MFS scores indicating worse expectations about future memory proficiency (df = 66, F = 2.6, *p* = 0.10), and an overall significantly lower domain score for self-rated memory appraisal (df = 66, F = 12.9, *p* = 0.001). After controlling for age, gender and education years, significant differences remained for subjective memory complaints (df = 63, F = 6.16, *p* = 0.016) and overall self-rated memory appraisal (df = 63, F = 9.0, *p* = 0.004).

### Objective cognitive performance

Executive function was generally superior in the HME group compared to LME, inclusive of tests of matrix reasoning (df = 66, F = 3.9, *p* = 0.05) and COWAT (df = 66, F = 6.7, *p* = 0.012) and non-significant trends were observed for faster task completion in TMTA (df = 66, F = 1.8, *p* = 0.179) and TMTB (df = 66, F = 2.1, *p* = 0.153). Without covariate adjustment, the total executive domain score for HME was significantly higher than LME (df = 66, F = 6.1, *p* = 0.016); after controlling for age, gender and education years, this comparison became non-significant (df = 63, F = 1.8, *p* = 0.181, Fig. 1). Memory function was significantly higher in the HME group than LME before (df = 66, F = 8.5, *p* = 0.005) and remained so after inclusion of the same covariate control (df = 63, F = 5.4, *p* = 0.024, Fig. 1)

### Hippocampus volume and morphology

Hippocampus volume was significantly larger in the HME as compared to the LME group, on both the left (df = 54, F = 4.8, *p* = 0.032) and right side (df = 54, F = 5.1, *p* = 0.027), reflected by a greater bilateral average hippocampi volume (df = 54, F = 8.1, *p* = 0.006, after adjustment for age, sex, education year and total intracranial volume Fig. 2). This finding was still significant after additionally controlling for hypertension status, occupational status, ADAS-Cog, CDR, physical activity and diabetes status (Supplementary).

Computational vertex analysis found that the morphology of the hippocampal surface was also significantly different between managerial groups. After controlling for sex and correction for multiple comparisons, those in the HME group had significantly larger volume (more volume of HME denoted by outwards inflation) at the lateral aspect of the anterior hippocampus (consistent with CA1 hippocampal subdivision) and the posterior tail (Fig. 2). The same regions were observed after additionally controlling for age and education years (Figure S1 in Supplementary).

### Hippocampal functional connectivity

Figure 3a shows the results of external erosion of the standard AAL hippocampal template in order to base our functional connectivity (FC) analysis on a hippocampal seed that takes into account atrophy of this structure. We separately tested right and left hippocampal-FC between HME and LME groups. For the right hippocampus seed, a one sample *t*-test illustrates the hippocampal FC map across the whole cohort (Fig. 3b, voxel-level p_(FWE-corr)_ = 0.000005, k > 200). Using a more rigid threshold, we found this FC map included biliateral hippocampi, parahippcampus, thalamus, putamen, precunues and middle cingulate cortex (Table S2 and Figure S2 in Supplementary). A two group *t*-test with sex, age and education as covariates found there was a significant difference at the right Prefrontal Cortex (rPFC, Brodman Area 10) after whole-brain correction (cluster-level p_(FDR-corr)_ = 0.022; k = 237; T = 3.99; [30 66 2]; Fig. 3c). LME had significantly higher FC between the right hippocampus and rPFC compared to the HME group controlling for age, sex and education years (F = 26.8, df = 59, *p* < 0.001, Fig. 3d). This finding was still significant after additionally controlling for hypertension status, occupational status, ADAS-Cog, CDR, physical activity and diabetes status (Supplementary). There was no difference for left hippocampal-FC after multiple comparison correction, though an identical left hippocampus and rPFC connection was observed using a lower threshold (Table S3 and Figure S3 in Supplementary). In general, right hippocampal-rPFC connectivity was negatively correlated with hippocampal volume (r = −0.330, *p* = 0.017, corrected for age, sex and education years Fig. 3e). Furthermore, this specific type of functional connectivity was correlated with the Self-rated Memory Appraisal domain (r = 0.420, *p* = 0.002, controlling for age, sex and education years Fig. 3f).

### A model of competing brain-behavior relationships

We built an initial model based upon significant unadjusted bivariate correlations, depicted by two-way arrows among the variables of self memory appraisal, executive domain and memory domain scores, hippocampal volume, functional connectivity between right hippocampus and right prefrontal cortex (rHP-rPFC), age and education years (*p* < 0.05, Supplementary, Figure S4). Table S4 (Supplementary) shows the zero-order (unadjusted) correlations between managerial experience and these variables of interest. In our subsequent models, directionality from hippocampal volume to memory performance was preset because structural brain changes evolve over a relatively long time periods (Zhang et al. 2011; Zheng et al. 2012). Directionality of other inter-variable associations was not possible to assign and are depicted by two-way arrows.

Inter-relationships between the variables were next explored through a series of hierarchical multiple regression analyses designed to develop the most parsimonious model. For all models, demographic information (i.e., sex, age and education years) as well as managerial experience were entered into the model as a first step with a backwards elimination criterion, and then all four remaining independent variables (IVs) were entered in a second backwards elimination step (Table S5, statistic details provided in Supplementary).

**Fig. 4:**
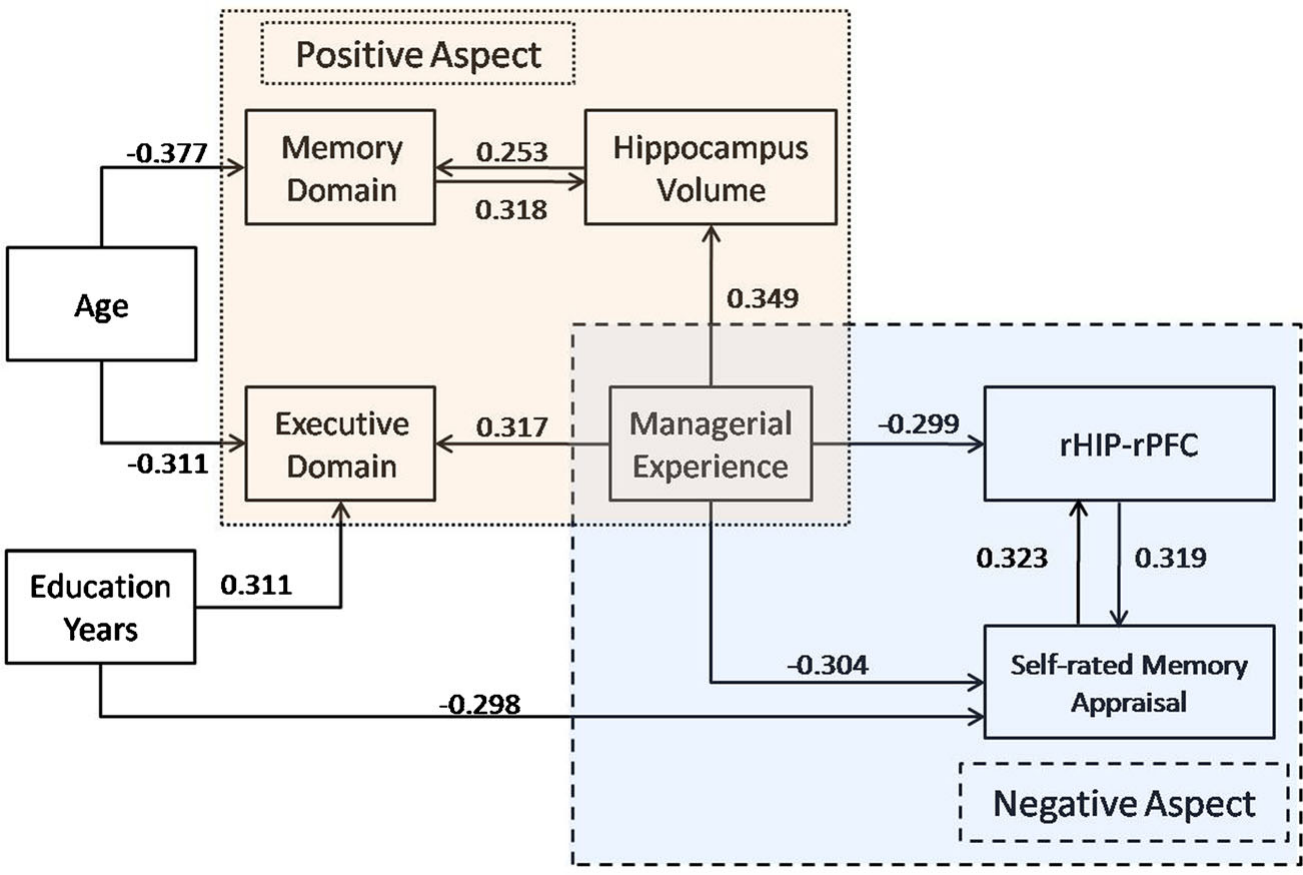
Simplified model of midlife managerial experience and independent links with age and late life hippocampal volume, cognition, self-rated memory appraisals and functional connectivity. The arrows pointed from independent variable to dependent variable, and the value was the Beta value in the regression model. See Supplementary for details of structural equation analyses used to build this model

Overall, the model suggested by these analyses is summarized in Fig. 4. Two major counter-veiling relationships were observed for midlife managerial experience. On the one hand, HME was related to better objectively-measured executive and memory performance, the latter mediated by protection of hippocampal volume. Formal mediator testing was completed using the Sobel test (Preacher and Hayes 2004). This test indicated that hippocampus volume positively mediates the relationship between managerial experience and objective memory performance (Mean = 0.751, 95%CI = [0.0067,0.1915]). On the other hand, HME also had a negative association with subjective memory appraisals, correlated with less hippocampal connectivity with mid-frontal cortex. We also tested a second formal mediating effect and found rHIP-rPFC functional connectivity positively mediates the link between managerial experience and self-rated memory appraisals (Mean = −0.2339, 95%CI = [−0.4296, −0.0552]). Age was negatively correlated with memory and executive performance, whereas education years were positively correlated with executive performance, but negatively related to self-rated memory appraisals. Multicollinearity was not a major issue in these models given a maximal variance inflation factor of 2.04 between managerial experience and memory performance.

## Comments

Managerial experience in midlife may exhibit powerful, competing and complex relationships with brain structure and function in late life. In a non-amnestic MCI cohort, we demonstrate for the first time a positive relationship between managerial experience and hippocampal volume. Preservation of hippocampal volume in HME group mediated their tendency to display superior memory function. Moreover, supervising workers was linked to differences in hippocampal morphology, reserving hippocampal volume in those subregions specifically vulnerable to neurodegeneration. These same individuals rated their own memory prospects worse, and these poor self-ratings were linked to lower hippocampal-prefrontal connectivity, a network implicated in self-perception. Management of large groups of employees during one’s working life may therefore benefit hippocampal structure and memory prowess in late life, but may link to poor subjective memory appraisal in late life and desynchronisation of related hippocampal networks.

Neuropathology and neuroimaging studies identify the hippocampus as one of the first brain regions affected in MCI and AD (Jack et al. 2010) (Braak and Braak 1991). For example, low hippocampal volume in non-demented elders independently predicts a 3-fold greater risk of incident dementia five years later (den Heijer et al. 2006). We found that the neuroprotective association between greater managerial experience and reduced hippocampal volume seen in healthy older adults (Suo et al. 2012) also appears to extend to those individuals with non-amnestic MCI. Similar cognitive lifestyle-related neuroprotective mechanism was recently reported on healthy (Arenaza-Urquijo et al. 2013) and AD cohorts (Shpanskaya et al. 2014) In contrast, the compensatory mechanism was supported by the reports of more global gray matter loss in elders with higher estimated cognitive reserve in amnestic MCI (Sole-Padulles et al. 2009), and AD patients (Seo et al. 2011) Further research is therefore required that contrasts cognitive lifestyle-related neuroprotective versus compensatory mechanisms in non-amnestic, amnestic MCI and AD.

Importantly, these neuroprotective findings appeared to be functionally relevant. In our hierarchical linear model, preserved hippocampal volume in HME group mediated a relative proficiency in memory tests. This is consistent with the functional role of this medial temporal lobe structure in memory networks (Ranganath and Ritchey 2012). Moreover, being in charge of a large group of workers was not only linked to gross volumetric advantages, but also subtle differences in morphometric patterns on the hippocampal surface. Participants with high-level managerial experience exhibited larger volume of the right CA1 hippocampal subregion, indicated by diminished involution of the lateral anterior body and hippocampal tail (Gerardin et al. 2009). These are the same regions specifically vulnerable to neurodegeneration in MCI and AD (Patenaude et al. 2011). In conjunction with our previous work (Suo et al. 2012), these findings suggest that employee supervision in midlife may help protect AD-susceptible hippocampal areas from neurodegeneration in later life, an effect that mediates a relative sparing of memory function in the setting of MCI. Multiple mechanisms, including both neuroprotective effects in the CA1 hippocampal subregion and cortical compensatory processes (Valenzuela et al. 2012) may therefore be implicated for explaining the strong and independent epidemiological link between work-related managerial experience and a 42 % lower risk for dementia (Schmand et al. 1997). Further, as explored in our previous work (Suo et al. 2012), managerial work is heavily reliant on cognitive processes such as linguistic competency, verbal comprehension and verbal memory (Boatsman et al. 1983; Christoffels et al. 2006) (Penley et al. 1991) (Bolton and Dewatripont 1994), processes that together may trigger long term neuroplastic and neuroprotective changes in the hippocampus. In addition, we previously found that managerial experience during working life tends to bias a person towards seeking out more social contacts in late life (Suo et al. 2012), a behavioral trait that is linked to lower dementia risk (Fratiglioni et al. 2000) and may protect the hippocampus (Fratiglioni et al. 2004). A similar result was again seen in this sample, and hence may represent a possible behavioral explanation for these long term associations.

Given these objective benefits, it was somewhat unexpected that occupational supervision was also linked to negative self-appraisal. Retired high-level managers exhibited consistently worse self-rated memory appraisals compared to the LME group. These ratings were assessed using the MARS-MFS, a sensitive instrument that asks individuals to predict their own future proficiency on day-to-day memory challenges, for example remembering a person’s name, being able to recount news stories, and following instructions of a short route. Whilst superior to their peers in the LME group on several memory tests, these individuals rated themselves 2.7-fold less capable. One possible technical explanation is that that MARS’ questions about predicted prospective memory focused on a memory domain that was simply not evaluated by our traditional objective memory tests, a question for future studies. Alternatively, individuals in HME may have dropped further from a higher premorbid level yet are still outperforming LME participants on objective tests, in effect a long term change that has triggered their concerns. Longitudinal data will be required to confirm this hypothesis. From a clinical perspective, since subjective memory complaint is a core criterion for MCI (irrespective of subtype), and is itself a significant independent predictor for incident dementia (Waldorff et al. 2012; Geerlings et al. 1999), ex-high level managers may be at higher risk for MCI by virtue of inaccurately low self-appraisals of their memory ability rather than memory impairment per se.

Interestingly, low self-appraisals in the HME group were associated with absent resting state functional connectivity between right hippocampus and right PFC, in contrast to positive connectivity and self-appraisals in LME group. There are a number of plausible reasons for why rHP-rPFC connectivity may potentially mediate these inter-relationships, suggested by the formal Sobel test of mediation. Firstly, right PFC (specifically BA10) has been heavily implicated in prospective memory paradigms (Burgess et al. 2007), which require a shift of perception from the immediate environment to an alternate imaged future environment inclusive of oneself (Buckner and Carroll 2007). The lateral part of BA10 has been specifically associated with delayed intentions and self-generated and self-maintained thought processes (Burgess et al. 2007). Secondly, the hippocampus and BA10 are both involved in decision-making tasks that require self appraisal (Schmitz and Johnson 2006). Thirdly, the structure of the default mode network at rest is similar to during self-projection processes such as *prospection* (the act of thinking about the future) (Buckner and Carroll 2007). The spontaneous right PFC -hippocampal connectivity we identified may potentially participate in a network related to mnemonic self-perception or prospective function. This hypothesis is consistent with our observation of an independent correlation between the strength of any such connectivity and better self-rated perceptions about future memory proficiency. Interestingly, Wang et al. (Wang et al. 2011) found decreased functional connectivity between the right hippocampus and prefrontal areas (BA10 and BA11) when comparing MCI participants to age-matched healthy participants. As such, deterioration of this functional network may be an early *non-cognitive* biomarker of dementia risk, linked in our study to low memory-related perceptions. Clearly, this hypothesis requires further research.

The most striking aspect of our findings was the independent and contrary relationship of midlife managerial experience on objective memory function and hippocampal structure on the one hand, and worse memory self-ratings and hippocampal-PFC connectivity on the other. These results are germane to the longstanding controversy over the relative importance of subjective versus objective memory in the older adult (Jorm et al. 1997) (Rabbitt and Abson 1991) (Perlmutter 1978). In the specific context of occupational history, a longitudinal study of retired managers noted that memory self-ratings were not correlated with age-related changes in objective memory performances (Poitrenaud et al. 1989). A large epidemiological study further suggests that the discrepancy between subjective and objective cognitive impairment was influenced by a person’s social environment – a wider social environment may lead to more opportunities in which cognitive function is ‘tested’ against peers and hence personally perceived (Trouton et al. 2006). Other explanations are possible, including ex-high level managers developing higher standards of self-assessment, a type of ‘cognitive hyper-vigilance’, or alternatively personality traits may interact with vocational choice and subsequent self-appraisals in the context of age-related change. Based on our data we can, however, exclude a number of potential explanations given there were no managerial group differences in depressive symptoms, general physical activity, socioeconomic status, retirement status, general cognitive lifestyle activity or presence of different cardiovascular risk factors.

This study also had some limitations. There was for example an imbalance in sub-group size in our cohort in favour of LME. This may of course be related to the protective effect of HME being linked to lower risk for dementia (Schmand et al. 1997), but our cross-sectional study cannot clarify the issue. Similarly, our model tested for cross-sectional associations between variables, and so the possibility of reverse causation or residual confounds cannot be excluded. For example, factors outside of our models (e.g., personality, early life activities or stress) may affect brain structure or function throughout the lifespan and may also be associated with managerial experience. Longitudinal data are needed to identify possible causal relationships. Also, our participants were non-amnestic MCI. Considering the aetiology of naMCI is even less well understood than aMCI and is likely to reflect both Alzheimer and non-Alzheimer pathology, the generalizability of these findings to amnestic MCI is not clear.

In conclusion, we found a clear double dissociation related to midlife managerial experience. On average, ex-high level managers had a larger and less AD-like hippocampus compared to individuals with low managerial experience, a difference that formally mediated superior mnemonic function. By contrast, they also made more pessimistic predictions about their own future memory proficiency, correlated to diminished hippocampal-prefrontal connectivity at rest. Hierarchical regression clarified these complex relationships, suggesting both positive and negative long term links between midlife managerial experiences and brain structure and function in late life.

## Electronic supplementary material

Below is the link to the electronic supplementary material.

ESM 1 (DOC 1238 kb)

ESM 2 (GIF 454 kb)
