# Supplementary material for: Midlife managerial experience is linked to late life hippocampal morphology and function
Source: Brain Imaging Behav. 2016 Nov 15;11(2):333–45. doi: 10.1007/s11682-016-9649-8 (PMC5408055; doi:10.1007/s11682-016-9649-8)
Supplement: Supplementary file 1 — (DOC 1238 kb) [file 11682_2016_9649_MOESM1_ESM.doc]

**SUPPLEMENTARY**

**Methods**

Socio-demographic, Health Status, Cognitive and Psychological Assessment

All socio-demographic and health status data were obtained by self-report using structured interviews. All cognitive and psychological data were acquired by an experienced neuropsychologist. For details of full neuropsychological battery and psychological test instruments see the SMART protocol . Tests reported upon here were:

A) Subjective Memory Complaint (SMC): study-specific questionnaire of seven simple binary questions (yes/now) related to current memory concerns. Items include noticing memory difficulties; concern about difficulties; duration of concern; other people noticing difficulties; informing others of concern; memory worse than peers; and seeking treatment. A total maximum score of 7 is possible with higher scores indicative of greater subjective difficulties.

B) Self-rated Memory Awareness: self-rated predictions of prospective memory competency using the Memory Awareness Rating Scale – Memory Function Scale (MARS - MFS) . The MARS-MFS was administered to assess participant ratings of their memory functioning in relation to aspects of daily living on a five-point scale very good to very poor function. Items include remembering names, remembering to attend appointments, being able to recall a news item, and recalling directions.

These two assessments were combined into a *Subjective Memory Appraisal* (*SMA*) domain score by averaging z-scores, referenced to whole baseline sample (N=100).

C) *Executive domain objective cognition*: average z-score (after reversing sign of test where appropriate and reference to whole sample) of Trail Making Test A (TMTA) time (reversed) , Trail Making Time B time (reversed) , WAIS III Matrices sub-test total correct , Controlled Oral Word Association Test (COWAT) total and WAIS III Similarities sub-test total correct .

MRI

MRI data were acquired on a Philip 3T Scanner. The scan parameters of T1 weighted structural MRI scans were: TR/TE=6.39/2.9ms, matrix size=256x256, 190 slices with 1mm slice thickness, no gap; resulting 1x1x1 mm^3^ isotropic voxels. Eye-closed resting-state functional MRI was collected after structural scan with parameters: TR/TE=2000/30ms, 28 slices with 4.5 mm slice thickness, no gap, 200 volumes.

Structural MRI Analysis

Firstly, raw T1-weighted MRI scans were checked for obvious anatomical or positional abnormalities. Secondly, a brain mask was generated for each individual participant using SPM8 (Statistical Parametric Mapping, Wellcome Department of Imaging Neuroscience, London, UK). For each individual, the brain was extracted by applying their brain mask on to the original image. Manual checking was performed to check against errors.

Left and right hippocampi were segmented using the Oxford Centre for Functional MRI of the Brian (FMRIB)'s Integrated Registration and Segmentation Tool (FIRST) in FMRIB's Software Library (FSL) version 4.1.8. FIRST is a model-based procedure for subcortical segmentation based on prior knowledge gained from a large number of manually segmented T1-weighted images . We used the same method as previously applied to older individuals . Furthermore, we used the FIRST quality checking protocol established by the ENIGMA consortium (Enhancing Neuro-Imaging Genetics Through Meta-Analysis).

In brief, a standard spatial template (Montreal Neurological Institute, MNI) was affine co-registered to the original image and co-registration results individually manually checked. For each individual, left and right hippocampi were then segmented, followed by automated calculation of volumes and construction of a vertex/mesh model. Outliners were identified and visually checked for segmentation error. Nine participants were excluded from this analysis because segmentation failed due to abnormal brain structure or movement artefacts, so for this analysis N=59. Two group t-tests were performed based on vertex-to-vertex analyses on the both left and right hippocampus, with sex as a covariate. Results were corrected for multiple comparison errors and only significant vertexes with p-value<0.05 are reported after FDR correction.

Resting State Functional MRI analysis

Three functional MRI data had to be removed due to the artefacts, leaving N=64 for functional MRI analysis. fMRI data were preprocessed using SPM8-based DPARSF tool-box ([www.restfmri.net/](http://www.restfmri.net/)) based on published protocols . In brief, this involved: discarding the first ten volumes of each participant, slice timing, normalization to standard MNI space, re-sampling into 2x2x2 mm^3^ isotropic voxels, smoothing using a 8-mm kernel, removal of global signal trends, bypass filtering of 0.01-0.08Hz, and finally regressing out nuisance signals related to white matter, whole-brain, and CSF signal as well as 6 co-registration factors.

Hippocampus seed-wise functional connectivity maps were generated individually using Resting-State fMRI Data Analysis Toolkit (REST, www.restfmri.net/) software running on Matlab . Bilateral hippocampal masks were first selected from the Anatomical Automatic Labeling (AAL) template. Since individuals with MCI experience significant atrophy , the AAL hippocampus template was eroded externally by a 2mm kernel (using FSL), resulting in a core hippocampal template less likely to be sensitive to partial volume effects on the BOLD signal (**Fig. 5A**). Individual functional connectivity (FC) maps for the left and right hippocampus were then generated based on correlations between the mean signal timecourse within each hippocampal seed region and the rest of the brain. Voxelwise correlations were transformed into z-scores.

Post-processing was performed using SPM8. We tested the difference of hippocampal FC maps at a whole brain voxel-level, using a two-group t-test, controlling for age, sex and education years. Left and right hippocampus FC maps were tested separately. Cluster-level FDR correction was set at p<0.05, with an initial uncorrected p-value of 0.005 and voxel threshold of 15. After identification of significant cluster differences between groups, the mean correlation of this cluster was calculated for each individual, corresponding to the spontaneous state of functional connectivity between this cluster and the hippocampus.

Statistical Analysis and Modelling

All statistical analyses were completed using SPSS 21. Levene’s Tests were performed to test the equality of variance, due to an unequal sample size between high and low managerial experience groups. No significant results were found, indicating the between group analyses are statistically robust.

To generate the zero-order structural models, bivariate (Pearson’s) correlations were performed for normally distributed variables. For categorical variables (i.e. managerial experience), nonparametric Spearman’s correlations were used. Partial correlations were used to examine relationships after controlling for the covariates. The final model was based on a series of hierarchical linear regression analyses using backwards elimination to clarify the relationship between competing independent variables and the dependent variable.

Where appropriate, we explicitly tested for mediation effects using the Sobel test . The Sobel test formally evaluates the total, indirect and direct influence of a proposed causal variable X on outcome variable Y through mediator variable M. Key work by Preacher and Hayes (Preacher and Hayes, 2004) now means that the Sobel procedure (with bootstrap confidence intervals) can be implemented in SPSS.

**Results**

Analysis on MR results with additional covariance

In additional to control for age, gender, education year and TIV (for structural MR results only), we conducted the between group t-tests correcting for additional covariance.

For bilateral average hippocampi volume, we still find the significant different result after additionally adjusting for hypertension status (df=53, F=9.3, p<0.005), or occupational status (df=53, F=7.68, p=0.008), or ADAS-Cog, CDR, physical activity, hypertension status and diabetes (df=49, F=5.5, p=0.024).

For functional connectivity between right hippocampal and right prefrontal cortex, the significant group difference is still observed after additionally adjusting for objective memory performance (df=46, F=24.3, p=0.001) or occupational status (df=46, F=32.2, p<0.001), or ADAS-Cog, CDR, physical activity, hypertension status and diabetes (df=42, F=16.2, p<0.001).

Morphometric analysis with additional covariance.

Same morphometric analysis as main results was conducted using the identical input data, while additionally controlling for age and education years. After correcting for multiple comparison using randomise permutations (n=5000) with threshold-free cluster enhancement, we still find the significant regions (**Figure S1**) at similar location as **Figure 2C** on right hippocampus.


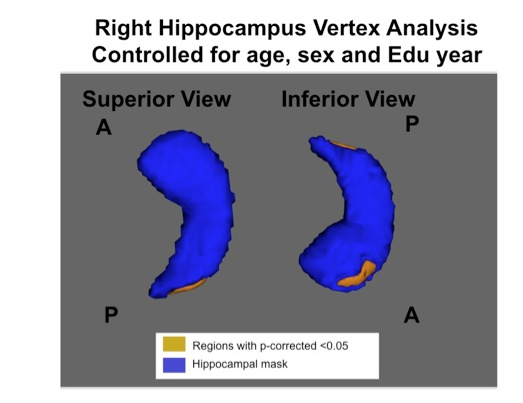


**Figure S1,** morphometric differences of right hippocampus between HME and LME group, significant areas had corrected for multiple comparison using permutation test (n=5000, threshold-free cluster enhancement, p<0.05)

One sample t-test on right hippocampus seed FC maps

The right hippocampus see FC map was dominated globally over the brain, we further applied a threshold (t>9.5, p_FWE-corrected_ < 0.00000005, k>200) to break down the massive cluster into small regions indicating the core part of this right hippocampal FC network. Three clusters were founded at (a) bilateral hippocampus/parahippocampus, thalamus and putamen, (b) bilateral precuneus and (c) bilateral middle cingulate cortex (**Figure S2** and **Table S2**). No anti-correlated regions were found after multiple comparison corrections.

The FC between right HP and other subcortical regions including thalamus may reflect to hippocampal- anterior thalamic pathway, which related to memory . Right hippocampus also functional synchronized with precuneus and middle cingulate cortex. As these regions are also hubs of DMN, this result is in line with the literature that HP is part of the DMN network .

*
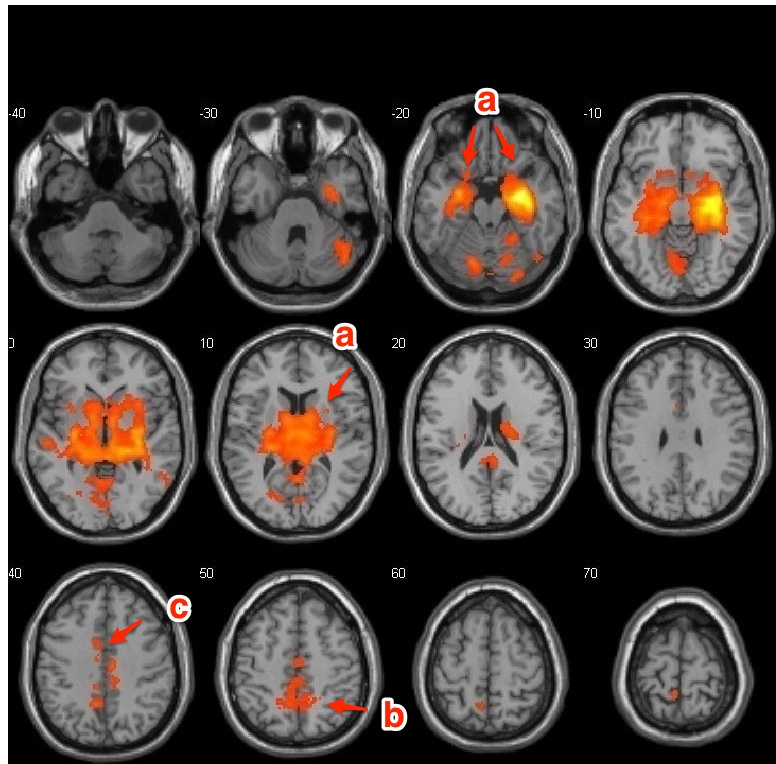
*

**Figure S2**, one sample t-test of right hippocampus seed FC maps showed the core functional connectivity regions at thalamus (a), putamen (a), parahippocampus (a), precuneus (b), and middle cingulate cortex (c), threshold at t>9.5, p_FEW-corrected_<0.0000005, and cluster size>200. No anti-correlated areas were observed after multiple comparison correction.

| **Region** | **MNI coordinates** | | | **Cluster Size** | **P_correction_** | **t-Value** |
| --- | --- | --- | --- | --- | --- | --- |
|  | X | Y | Z |  |  |  |
| Bilateral hippocampus, parahippocampus, thalamus putamen (cluster a) | -28 | -12 | -16 | 16k | <0.001 | 31.90 |
|  | 36 | -16 | -16 |  |  | 30.75 |
|  | 36 | -26 | -14 |  |  | 21.44 |
| Bilateral precuneus (cluster b) | -6 | -52 | 44 | 952 | <0.001 | 12.25 |
|  | -4 | -42 | 68 |  |  | 11.44 |
|  | -6 | -50 | 66 |  |  | 11.22 |
| Bilateral middle cingulate cortex (cluster c) | 8 | -14 | 40 | 319 | <0.001 | 11.55 |
|  | -4 | -10 | 46 |  |  | 10.71 |
|  | -6 | 12 | 34 |  |  | 10.61 |

**Table S2**. One sample t-test of right HP FC maps across all cohort (p_FWE-corrected_<5e-8, Cluster size>200)

Marginal group difference between LME and HME on left hippocampus FC maps

We applied a less rigid threshold on two-group comparison of left hippocampus functional connectivity (i.e., p_uncorrected_<0.01); and found the similar finding as right hippocampus seed but not significant after correction. In general, high managerial experience group exhibited reduced hippocampal connectivity at several regions comparing with low managerial experience. Importantly, the right prefrontal cortex cluster (cluster b in **Figure S3**) in figure below is at the identical location of the results of right hippocampus FC (**Figure 3-C**). A couple of more clusters were found at left inferior frontal lobe (cluster a) and left medial superior frontal lobe (cluster c). However, none of these clusters survived after multiple comparison correction. Further, there is no significant high FC in favour of HME. Cluster details (**Table S3**) were attached.


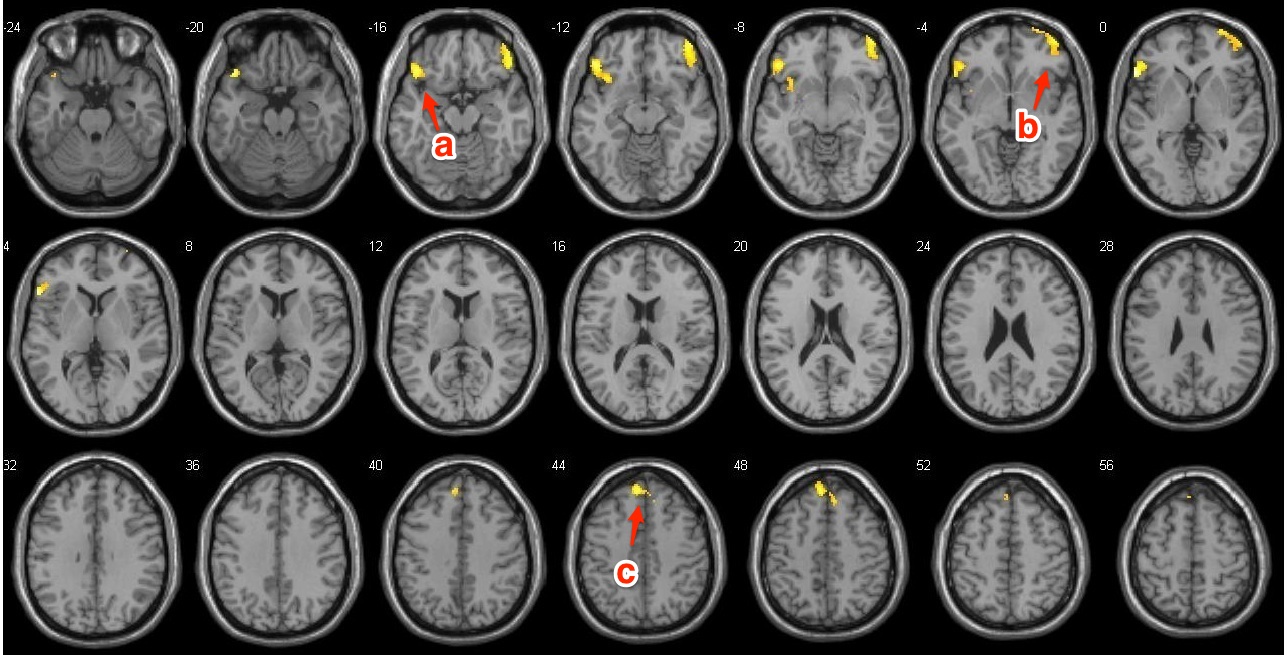


**Figure S3,** left hippocampus FC difference of Low ME versus High ME. Clusters were threshold at p_uncorrected_ < 0.05, cluster size > 50, but not survived after multiple comparison correction. Hot spots indicated high-level FC of left hippocampus of LME over HME, no high FC in favour of HME was found.

| **Region** | **MNI coordinates** | | | **Cluster Size** | **P_correction_** | **t-Value** |
| --- | --- | --- | --- | --- | --- | --- |
|  | X | Y | Z |  |  |  |
| Left inferior frontal lobe (cluster a) | -56 | 26 | 0 | 478 | 0.332 | 4.50 |
|  | -42 | 26 | -18 |  |  | 3.80 |
|  | -48 | 34 | -12 |  |  | 3.74 |
| Right prefrontal cortex (cluster b) | 44 | 56 | -8 | 444 | 0.398 | 3.32 |
|  | 44 | 46 | -10 |  |  | 3.13 |
|  | 48 | 38 | -16 |  |  | 3.10 |
| Left medial superior frontal lobe (cluster c) | -6 | 46 | 44 | 171 | 0.987 | 3.64 |
|  | 10 | 36 | 48 |  |  | 2.80 |
|  | -4 | 38 | 54 |  |  | 2.65 |

**Table S3.** Left Hippocampus FC difference between High ME and Low ME (p_uncorrected_<0.05, Cluster size>50)

Statistical details of hierarchical multiple regression analysis

**Table S4.** Bivariate correlations between key variables of interest.

|  | | Managerial Experience | Self Memory Appraisal | Executive Domain | HV | rHP-rPFC | Memory Domain | Age | Education |
| --- | --- | --- | --- | --- | --- | --- | --- | --- | --- |
| Managerial Experience**^** | | **1** | **-0.399*** | **0.28*** | **0.288*** | **-0.38**** | **0.291*** | -0.192 | 0.161 |
| Self Memory Appraisal | |  | 1 | 0.02 | -0.079 | **0.405**** | 0.22 | -0.14 | -0.200 |
| Executive Domain | |  |  | 1 | 0.092 | -0.104 | **0.443**** | **-0.353**** | **0.453**** |
| HV | |  |  |  | 1 | **-0.294*** | **0.35*** | -0.249 | 0.075 |
| rHP-rPFC | |  |  |  |  | 1 | 0.024 | 0.152 | **-0.273*** |
| Memory Domain | |  |  |  |  |  | 1 | **-0.396**** | **0.316*** |
| Age | |  |  |  |  |  |  | 1 | -0.152 |
| Education | |  |  |  |  |  |  |  | 1 |
|  | *p<0.05; **p<0.01; **^**Non parametric Spearman’s correlation coefficients are presented along the first row. Pearson correlation coefficients listed for other correlations. HV = average of bilateral hippocampal volume; rHP-rPFC = functional connectivity between resting state right hippocampus and right middle prefrontal cortex activity. | | | | | | | | |


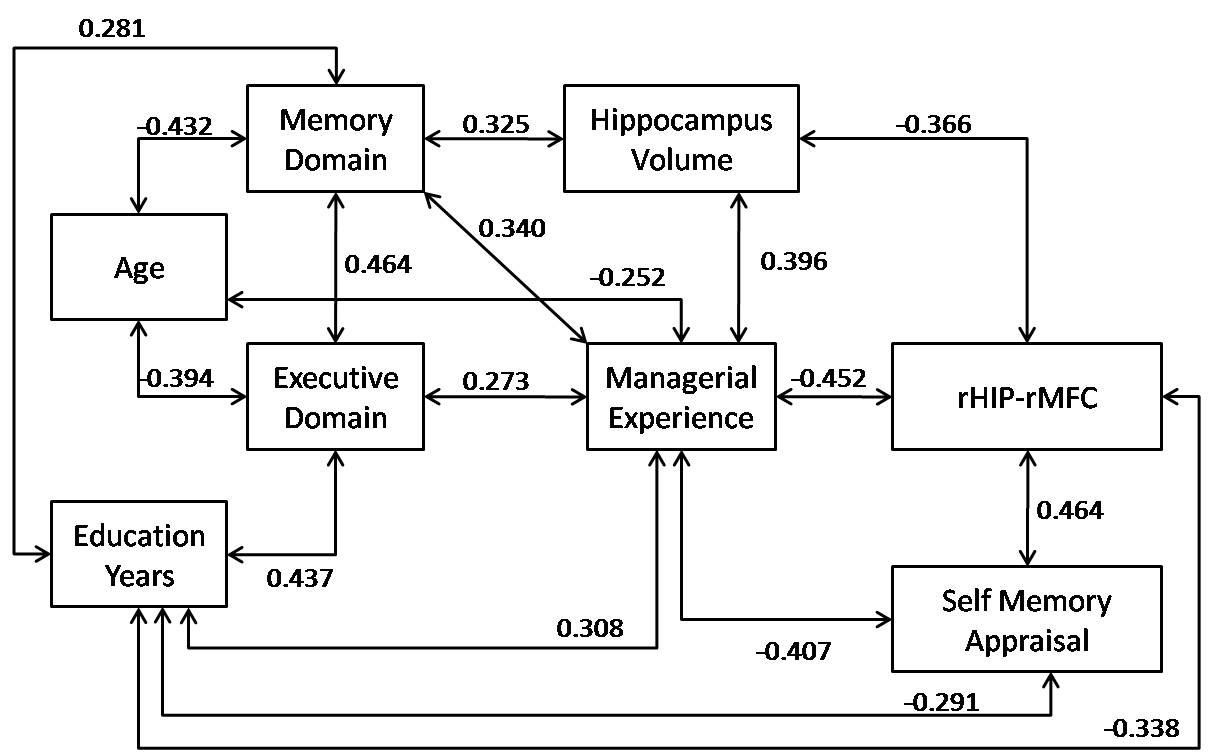


**Figure S4.** Initial unadjusted correlational model. Weightings for each pathway indicate bivariate correlation coefficient.

In Model A (**Table S5, Supplementary**) function connectivity between the right hippocampus and right PFC was the dependent variable (DV). Age and education level (years) were eliminated from the model; memory performance, executive function, sex and hippocampal volume had no significant association. Managerial experience and self-rated memory appraisals both had significant relationships with the DV. Model B (**Table S5**) tested predictors of self-rated memory appraisals. Managerial experience had a significant negative relationship, whereas hippocampal functional connectivity and education was a positive predictor. Model C tested predictors of memory domain performance (DV). There were two IVs with significant relationships in the final model, age as a negative IV and hippocampal volume as a positive IV. Model D tested predictors of executive function (DV). Age, sex and managerial experience survived to the final model. Model E tested a model of hippocampal volume (DV). The final IVs were managerial experience and memory performance.

**Table S5.**

| A. Dependent Variable: rHP-rPFC | | | | |
| --- | --- | --- | --- | --- |
| Initial Model | | Final Model | | |
|  |  | R square | F | p-value |
|  |  | 0.348 | 5.802 | **<0.001** |
|  | | Beta | t | p-value |
| Step 1 | 1.Sex | -0.117 | -1.042 | 0.303 |
|  | 2. Managerial Experience | -0.299 | 2.008 | **0.05** |
|  | 3. Age | *Excluded* |  |  |
|  | 4. Education Level | *Excluded* |  |  |
|  |  |  |  |  |
| Step 2 | 1. Executive Domain | -0.113 | -0.861 | 0.393 |
|  | 2. Hippocampal Volume | -0.222 | 1.726 | 0.091 |
|  | 3. Self Memory Appraisal | 0.323 | 2.528 | **0.015** |
|  | 4. Memory Domain | 0.100 | 0.769 | 0.446 |

| B. Dependent Variable: Self Memory Appraisal | | | | |
| --- | --- | --- | --- | --- |
| Initial Model | | Final Model | | |
|  |  | R square | F | p-value |
|  |  | 0.319 | 4.611 | **0.001** |
|  | | Beta | t | p-value |
| Step 1 | 1.Sex | 0.111 | 0.957 | 0.343 |
|  | 2. Managerial Experience | -0.304 | -1.990 | **0.05** |
|  | 3. Age | *Excluded* |  |  |
|  | 4. Education level | -0.298 | -2.208 | **0.032** |
|  |  |  |  |  |
| Step 2 | 1. Executive Domain | 0.253 | 1.834 | 0.073 |
|  | 2. Hippocampal Volume | 0.128 | 0.897 | 0.375 |
|  | 3. rHP-rPFC | 0.319 | 2.277 | **0.027** |
|  | 4. Memory Domain | 0.079 | 0.595 | 0.555 |

| C. Dependent Variable: Memory Domain | | | | |
| --- | --- | --- | --- | --- |
| Initial Model | | Final Model | | |
|  |  | R square | F | p-value |
|  |  | 0.318 | 5.201 | **<0.001** |
|  | | Beta | t | p-value |
| Step 1 | 1.Sex | *Excluded* |  |  |
|  | 2. Managerial Experience | *Excluded* |  |  |
|  | 3. Age | -0.377 | -2.916 | **0.005** |
|  | 4. Education Level | 0.142 | 1.021 | 0.312 |
|  |  |  |  |  |
| Step 2 | 1. Executive Domain | 0.176 | 1.241 | 0.220 |
|  | 2. Hippocampal Volume | 0.253 | 2.037 | **0.047** |
|  | 3. rHP-rPFC | 0.075 | 0.533 | 0.597 |
|  | 4. Self Memory Appraisal | 0.103 | 0.754 | 0.455 |

| D. Dependent Variable: Executive Domain | | | | |
| --- | --- | --- | --- | --- |
| Initial Model | | Final Model | | |
|  |  | R square | F | p-value |
|  |  | 0.367 | 5.469 | **<0.001** |
|  | | Beta | t | p-value |
| Step 1 | 1.Sex | *Excluded* |  |  |
|  | 2. Managerial Experience | 0.317 | 2.161 | **0.036** |
|  | 3. Age | -0.311 | -2.426 | **0.019** |
|  | 4. Education Level | 0.311 | 2.432 | **0.019** |
|  |  |  |  |  |
| Step 2 | 1. Memory Domain | 0.141 | 1.020 | 0.313 |
|  | 2. Hippocampal Volume | -0.223 | -1.755 | 0.086 |
|  | 3. rHP-rPFC | -0.089 | -0.631 | 0.531 |
|  | 4. Self Memory Appraisal | 0.251 | 1.864 | 0.069 |

| E. Dependent Variable: Hippocampal Volume | | | | |
| --- | --- | --- | --- | --- |
| Initial Model | | Final Model | | |
|  |  | R square | F | p-value |
|  |  | 0.239 | 4.39 | **0.002** |
|  | | Beta | t | p-value |
| Step 1 | 1.Sex | *Excluded* |  |  |
|  | 2. Managerial Experience | 0.349 | 2.196 | **0.033** |
|  | 3. Age | *Excluded* |  |  |
|  | 4. Education Level | *Excluded* |  |  |
|  |  |  |  |  |
| Step 2 | 1. Memory Domain | 0.318 | 2.407 | **0.02** |
|  | 2. Executive Domain | -0.233 | -1.663 | 0.099 |
|  | 3. rHP-rPFC | -0.249 | -1.663 | 0.103 |
|  | 4. Self Memory Appraisal | 0.112 | 0.770 | 0.445 |

**Supplementary References**

Aggleton, J.P., O'Mara, S.M., Vann, S.D., Wright, N.F., Tsanov, M., Erichsen, J.T. 2010. Hippocampal-anterior thalamic pathways for memory: uncovering a network of direct and indirect actions. Eur J Neurosci 31(12), 2292-307.

Chao-Gan, Y., Yu-Feng, Z. 2010. DPARSF: A MATLAB Toolbox for "Pipeline" Data Analysis of Resting-State fMRI. Frontiers in systems neuroscience 4, 13.

Clare, L., Wilson, B.A., Carter, G., Roth, I., Hodges, J.R. 2002. Assessing awareness in early-stage Alzheimer's disease: Development and piloting of the Memory Awareness Rating Scale. Neuropsychological Rehabilitation 12(4), 341-62.

Erickson, K.I., Prakash, R.S., Voss, M.W., Chaddock, L., Hu, L., Morris, K.S., White, S.M., Wojcicki, T.R., McAuley, E., Kramer, A.F. 2009. Aerobic fitness is associated with hippocampal volume in elderly humans. Hippocampus 19(10), 1030-9.

Erickson, K.I., Voss, M.W., Prakash, R.S., Basak, C., Szabo, A., Chaddock, L., Kim, J.S., Heo, S., Alves, H., White, S.M., Wojcicki, T.R., Mailey, E., Vieira, V.J., Martin, S.A., Pence, B.D., Woods, J.A., McAuley, E., Kramer, A.F. 2011. Exercise training increases size of hippocampus and improves memory. Proc Natl Acad Sci U S A 108(7), 3017-22.

Gates, N.J., Valenzuela, M., Sachdev, P.S., Singh, N.A., Baune, B.T., Brodaty, H., Suo, C., Jain, N., Wilson, G.C., Wang, Y., Baker, M.K., Williamson, D., Foroughi, N., Fiatarone Singh, M.A. 2011. Study of Mental Activity and Regular Training (SMART) in at risk individuals: a randomised double blind, sham controlled, longitudinal trial. BMC Geriatr 11, 19.

Greicius, M.D., Krasnow, B., Reiss, A.L., Menon, V. 2003. Functional connectivity in the resting brain: a network analysis of the default mode hypothesis. Proc Natl Acad Sci U S A 100(1), 253-8.

Greicius, M.D., Srivastava, G., Reiss, A.L., Menon, V. 2004. Default-mode network activity distinguishes Alzheimer's disease from healthy aging: evidence from functional MRI. Proc Natl Acad Sci U S A 101(13), 4637-42.

Mitrushina, M., Boone, K.B., Razani, J., D'Elia, L.F. 2005. Handbook of Normative Data for Neuropsychological Assessment. Oxford University Press, USA.

Patenaude, B., Smith, S.M., Kennedy, D.N., Jenkinson, M. 2011. A Bayesian model of shape and appearance for subcortical brain segmentation. Neuroimage 56(3), 907-22.

Preacher, K.J., Hayes, A.F. 2004. SPSS and SAS procedures for estimating indirect effects in simple mediation models. Behavior research methods, instruments, & computers : a journal of the Psychonomic Society, Inc 36(4), 717-31.

Shi, F., Liu, B., Zhou, Y., Yu, C., Jiang, T. 2009. Hippocampal volume and asymmetry in mild cognitive impairment and Alzheimer's disease: Meta-analyses of MRI studies. Hippocampus 19(11), 1055-64.

Song, X.W., Dong, Z.Y., Long, X.Y., Li, S.F., Zuo, X.N., Zhu, C.Z., He, Y., Yan, C.G., Zang, Y.F. 2011. REST: a toolkit for resting-state functional magnetic resonance imaging data processing. PLoS One 6(9), e25031.

Spreen, O., Strauss, E. 1998. A Compendium of Neuropsychological Tests:Administration, Norms, and Commentary: Administration, Norms, and Commentary. Oxford University Press, USA.

Stein, T., Moritz, C., Quigley, M., Cordes, D., Haughton, V., Meyerand, E. 2000. Functional connectivity in the thalamus and hippocampus studied with functional MR imaging. AJNR Am J Neuroradiol 21(8), 1397-401.

Tzourio-Mazoyer, N., Landeau, B., Papathanassiou, D., Crivello, F., Etard, O., Delcroix, N., Mazoyer, B., Joliot, M. 2002. Automated anatomical labeling of activations in SPM using a macroscopic anatomical parcellation of the MNI MRI single-subject brain. Neuroimage 15(1), 273-89.

Wechsler, D. 1997. Wechsler Adult Intelligence Scales 3rd Edition. Harcourt Brace & Company.

Winkler, A.M., Ridgway, G.R., Webster, M.A., Smith, S.M., Nichols, T.E. 2014. Permutation inference for the general linear model. Neuroimage 92, 381-97.
