# Supplementary figures and images for: Midlife managerial experience is linked to late life hippocampal morphology and function
Source: Brain Imaging Behav. 2016 Nov 15;11(2):333–45. doi: 10.1007/s11682-016-9649-8 (PMC5408055; doi:10.1007/s11682-016-9649-8)

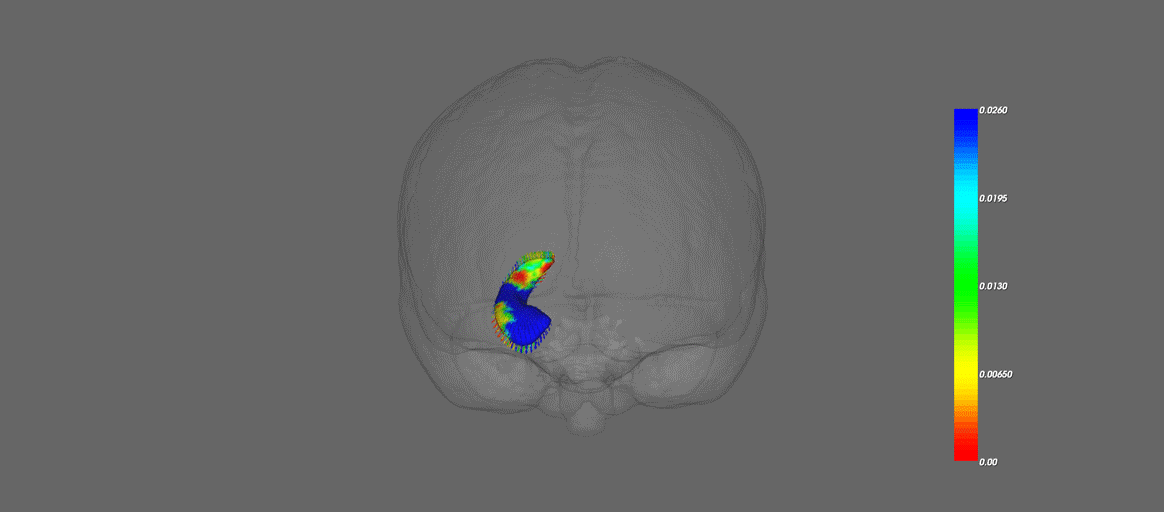

Supplement: Supplementary file 2 — (GIF 454 kb) [file 11682_2016_9649_MOESM2_ESM.gif]
